# Depth-dependent transcriptomic response of diatoms during spring bloom in the western subarctic Pacific Ocean

**DOI:** 10.1038/s41598-019-51150-8

**Published:** 2019-10-10

**Authors:** Shigekatsu Suzuki, Takafumi Kataoka, Tsuyoshi Watanabe, Haruyo Yamaguchi, Akira Kuwata, Masanobu Kawachi

**Affiliations:** 1 Center for Environmental Biology and Ecosystem Studies, National Institute for Environmental Studies, 16-2 Onogawa, Tsukuba, Ibaraki Japan; 2 Faculty of Marine Science and Technology, Fukui Prefectural University, 1-1 Gakuen-cho, Obama, Fukui Japan; 3 Tohoku National Fisheries Research Institute, Japan Fisheries Research and Education Agency, 3-27-5 Shinhama-cho, Shiogama, Miyagi Japan

**Keywords:** Microbial ecology, Marine biology

## Abstract

Diatoms play important roles in primary production and carbon transportation in various environments. Large-scale diatom bloom occurs worldwide; however, metabolic responses of diatoms to environmental conditions have been little studied. Here, we targeted the Oyashio region of the western subarctic Pacific where diatoms bloom every spring and investigated metabolic response of major diatoms to bloom formation by comparing metatranscriptomes between two depths corresponding to different bloom phases. *Thalassiosira nordenskioeldii* and *Chaetoceros debilis* are two commonly occurring species at the study site. The gene expression profile was drastically different between the surface (late decline phase of the bloom; 10 m depth) and the subsurface chlorophyll maximum (SCM, initial decline phase of the bloom; 30 m depth); in particular, both species had high expression of genes for nitrate uptake at the surface, but for ammonia uptake at the SCM. Our culture experiments using *T. nordenskioeldii* imitating the environmental conditions showed that gene expression for nitrate and ammonia transporters was induced by nitrate addition and active cell division, respectively. These results indicate that the requirement for different nitrogen compounds is a major determinant of diatom species responses during bloom maturing.

## Introduction

Diatoms contribute up to 20–40% of oceanic primary productivity forming large-scale blooms mainly in coastal and upwelling regions^1–3^. They are key organisms for carbon flux in the ocean, acting as large carbon sinks by vertical carbon transportation by sinking to deeper layers^4^. Several tens of species occur in diatom blooms; however, a few species are usually dominant (60–90% abundance). For example, *Chaetoceros* spp. and *Thalassiosira* spp. are dominant in the Oyashio region of the western North Pacific^5,6^ and the world ocean^7^.

Diatom blooms are induced by the supply of resources that promote growth, such as light, macronutrients, and iron. Water column stability and local mixing are indicated as factors promoting diatom blooms^8^. Increase of light intensity causes diatom blooms in coastal regions, such as the Narragansett Bay^9^, the fjord of western Norway^10^, and the Southern Ocean^11^. Diatoms are known to response to upwelled nutrients and grow rapidly^12,13^. Iron enrichment experiments, such as the western North Pacific^14^, suggest that supply of iron is an important factor of blooming of diatoms^15^. However, details of physiological processes in blooming diatoms, e.g., nutritional utilizations and cell responses of bloom-forming species, remain unclear.

Metatranscriptome analyses are useful for studying algal cell responses in natural environments^16–18^. Alexander *et al*.^19^ compared gene expression patterns of two bloom-forming diatoms in Narragansett Bay in the western North Atlantic and found differential gene expression in nitrogen and phosphorus metabolism pathways among the diatoms. However, there are methodological difficulties to applying metatranscriptome analysis to natural microbial communities. For example, sampling without disturbing transcripts is difficult because transcripts are susceptible to environmental change during sampling. Lack of reference genome information in public databases also prevents the selection of appropriate control organisms.

The western subarctic Pacific is a preferable field to study cell responses of bloom-forming species because large-scale diatom blooms occur annually in spring in this region^5,20,21^. The hydrography of this region in spring is complex, with dominant water columns: Oyashio Water (OW), Coastal Oyashio Water (COW), and modified Kuroshio Water (MKW)^22^. Warming by increasing solar radiation and inflow of the low-salinity COW lead to formation of a shallow surface layer and make phytoplankton use nutrients supplied by winter mixing to form massive diatom blooms^21–24^. The alleviation of light limitation due to the mixed layer shoaling and the accumulation of diatom cells due to the weakening of turbulence are also related to onset of diatom blooms^25^.

Here, we performed metatranscriptome analyses of diatom communities during spring blooms in the western North Pacific to reveal bloom-specific cell responses, focusing on differences in depth. We compared two diatom communities obtained from the sea surface (late decline phase of the bloom; 10 m) and subsurface chlorophyll maximum (SCM, initial decline phase of the bloom; 30 m), particularly targeting *T. nordenskioeldii* and *C. debilis*, which commonly occur during spring bloom in this region^21^. Moreover, gene expression analysis of nutrient-controlled cultures was conducted to confirm the metatranscriptomic analysis.

## Results and Discussion

### Characterization of the environments and diatom blooms

**Figure 1:**
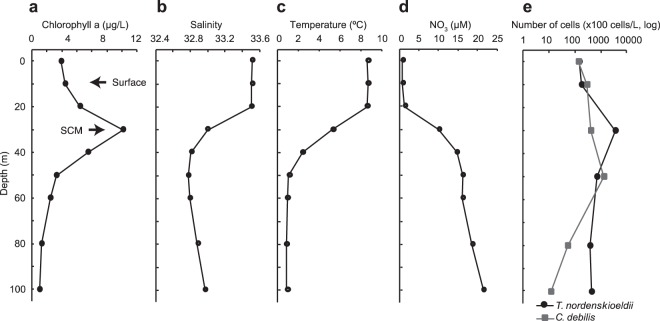
Features of the environment and diatom blooms. Concentration of chlorophyll *a* (**a**), salinity (**b**), temperature (**c**), nitrate (**d**) along depth are shown. The chlorophyll *a* concentration peaks at around 30 m, the subsurface chlorophyll maximum (SCM). The sample at 10 m was used as the surface sample. (**e**) Number of cells of *Tharassiosira nordenskioeldii* (black) and *C. debilis* (gray) along depth. The number of *T. nordenskioeldii* peaks at the SCM, but that of *C. debilis* gradually increases from the surface to 50 m depth.

We sampled an algal bloom at Station A4 located in the Oyashio region of the western subarctic Pacific. The SCM was observed at a depth of around 30 m. Here, the chlorophyll *a* concentration (12.1 µg L^−1^) was about three times higher than that at the sea surface (10 m depth; 4.4 µg L^−1^) (Fig. 1a; Supplementary Table S1). These chlorophyll levels are similar to the values observed during spring diatom blooms in this region^23,26^. Total cell numbers of diatoms were 275,830 and 1,600,465 cells L^−1^ in the surface and SCM, respectively. The environmental conditions between the surface and SCM were different in terms of temperature, salinity, and concentration of nitrate, nitrite, silicic acid, and phosphate (Fig. 1b–d; Supplementary Table S1). Temperature and salinity at the SCM (5.2 °C and 33.0) were lower than at the surface (8.7 °C and 33.5), indicating that the SCM and surface were affected by the water columns COW and MKW, respectively^27^. Upper layers including the layer of the surface sample (10 m) had lower nutrient concentrations, whereas lower layers, where the SCM sample was collected, had higher nutrient concentrations. Thus, the phytoplankton bloom we analysed would be in nutrient-rich conditions at the SCM (30 m) due to the influence of cold nutrient-rich COW, whereas low-nutrient conditions existed at the surface (10 m). To elucidate actively transcriptionally responding taxa, we performed taxonomic analyses of the RNA-seq reads (Supplementary Fig. S1). The RNA-seq reads were mainly composed of diatoms, Opisthokonta, Alveolata, and Viridiplantae both in the surface and SCM samples. Diatoms were the predominant group in both samples. The proportion of diatoms in the SCM layer (76.7%) was higher than that in the surface layer (51.4%), suggesting that diatoms have more active transcriptional responses in the SCM layer than at the surface. To elucidate development stages of the diatom blooms at the surface and SCM, we used an index, *I*_devel_^20^. *I*_devel_ was 0.95 and 0.77 at the surface and SCM, respectively, and thus diatoms of the surface and SCM are under late and initial decline phase of the diatom bloom. In the surface, nitrate concentration was lower than the SCM (Fig. 1d; Supplementary Table S1), however, the nitrogen concentration is not limiting factors for the diatom growth rates in the surface sample (Supporting Text; Supplementary Fig. S2). For light intensities, it has been reported that growth of *T. nordenskioeldii* is limited in cold water at high light intensity^28^. Thus, the cells in the surface can be affected by high light intensity.

Our direct microscopy counting showed that the main diatom species in the blooms were *T. nordenskioeldii*, *C. compressus*, *C. socialis*, *C. diadema*, *C. debilis*, and *Fragilariopsis cylindrus* (Supplementary Fig. S3a–e). RNA-seq or genome data of *F. cylindrus*, *C. socialis*, and *C. debilis* are available and fortunately, we could acquire the strain of *T. nordenskioeldii* from the same experiment region. Therefore, we selected those four diatoms as the representative transcripts in this study. Large cell numbers for *T. nordenskioeldii*, *C. socialis*, and *C. debilis* were also observed in the Oyashio region during spring diatom bloom^6,21^, and their relatives were observed in our past sampling in 2015 as described in Supporting Text and Supplementary Fig. S4.

### Overall gene expression patterns in *Thalassiosira nordenskioeldii* and *Chaetoceros debilis*

RNA-seq reads of both the surface and SCM samples were mapped to 91.6%, 59.0%, 6.0%, and 24.1% of all of the protein-coding genes originating from *T. nordenskioeldii*, *C. debilis*, *C. socialis*, and *F. cylindrus*, respectively. For more detail on the gene expression, the top two diatoms, *T. nordenskioeldii* and *C. debilis*, were subjected to DEG analysis because mapping rates of all replicates of *T. nordenskioeldii* and *C. debilis* were higher than *C. socialis*, and *F. cylindrus* (Supplementary Fig. S5). Few reads were mapped to *C. socialis*, which contradicted our microscopic cell counting showing that *C. socialis* was one of the major species (Supplementary Fig. S3a). This might be due to intraspecific diversity of the gene sequences^29^ and identification by microscopic observation is difficult to separate the cryptic genetic diversity.

**Figure 2:**
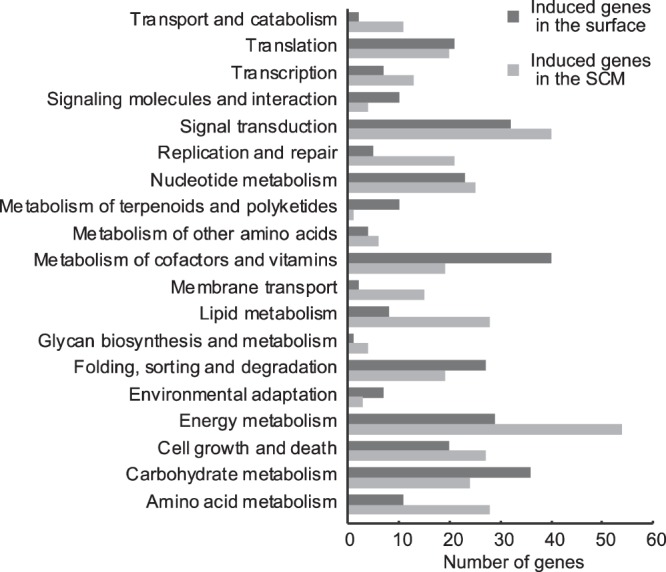
Functional classification of differentially expressed genes in *T. nordenskioeldii*. Functional analyses were performed using Kyoto Encyclopedia of Genes and Genomes (KEGG). Black and gray bars represent numbers of significantly induced genes (FDR < 0.01) at the surface and SCM, respectively.

Among the protein-coding genes, 2,882 and 542 genes of *T. nordenskioeldii* and *C. debilis*, respectively, were significantly differently expressed between the two depths (FDR < 0.01) (Supplementary Fig. S6a,b, Tables S2, S3. Higher gene expression was detected in the SCM sample (63.0% of total DEGs) for *T. nordenskioeldii* and in the surface sample (55.2% of total DEGs) for *C. debilis*. In addition, KEGG category-based functional analysis showed that genes for cofactors and vitamin metabolism dominated only in surface samples of *T. nordenskioeldii*, while genes for energy metabolism, lipid metabolism, and amino acid metabolism dominated only in the SCM (Fig. 2).

In the SCM, *T. nordenskioeldii* and *C. debilis* induced expression of some genes for S-G_2_/M phase-specific cyclin A and DNA replication system such as replication factor C and DNA helicase. In addition, expression of genes for silicon transporters (*SIT1*) were induced in the SCM samples (Supplementary Fig. S7). Silicon limitation alone induces transcription of *SIT1*^30^ and thus diatom cells at the SCM are under high silicon requirement. These results suggest that the diatoms in the SCM had more frequent cell division than the surface, which is consistent with our microscopic cell counting showing that the cell numbers of *T. nordenskioeldii* and *C. debilis* in the SCM samples were greater than those in the surface sample (Fig. 1e).

### Depth-dependent nitrogen metabolism in *Thalassiosira nordenskioeldii* and *Chaetoceros debilis*

**Figure 3:**
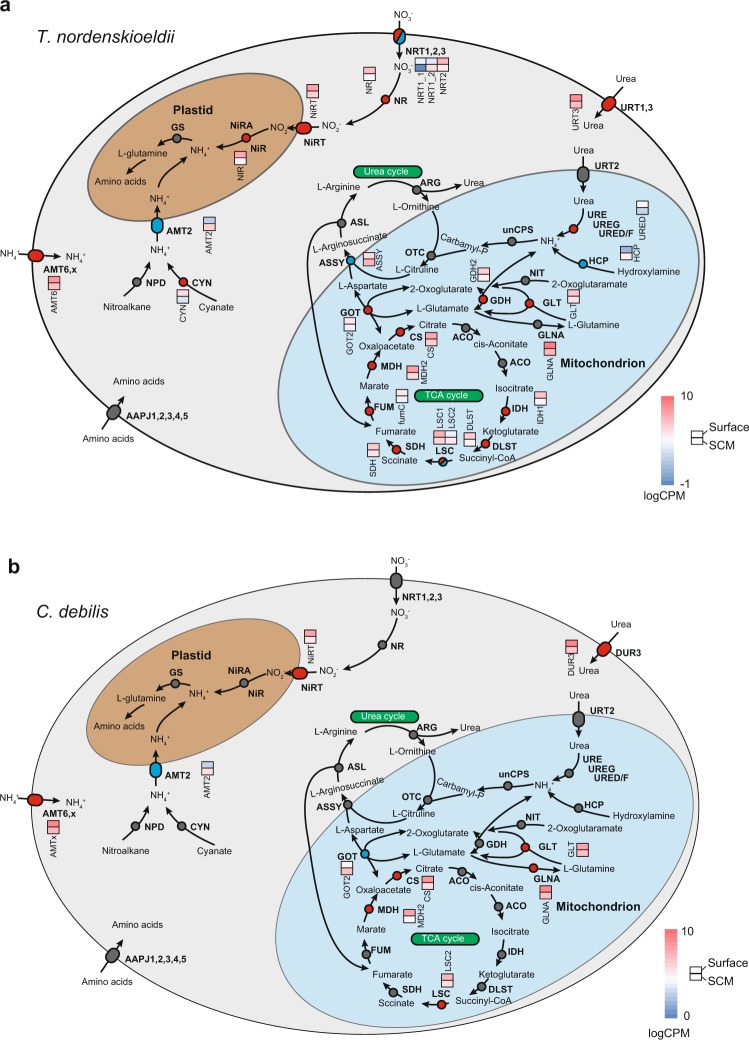
Metabolism map of nitrogen metabolism and the different gene expression patterns in bloom-forming diatoms. Metabolites, enzymes, and transporters of *T. nordenskioeldii* (**a**) and *C. debilis* (**b**) are shown in this figure. The names of enzymes (circles) and transporters (ovals) are shown in bold type. Genes induced at the surface and SCM are depicted as red- and blue-colored circles/ovals, respectively. For differently expressed genes (FDR < 0.01), heatmaps of relative expression level (logarithmic read count per million; RPM) are shown. AAPJ: General L-amino acid transport system, ACO: acyl-CoA oxidase, AMT: ammonium transporter, ARG: arginase, ASL: argininosuccinate lyase, ASSY: argininosuccinate synthase, CYN: cyanate hydratase, CS: citrate synthase, DLST: dihydrolipoamide S-succinyltransferase, DUR: urea transporter, FUM: fumarate hydratase, GS: glutamine synthase, GDH: glutamate dehydrogenase, GLNA: glutamine synthetase, GLT: glutamate synthase, GOT: aspartate aminotransferase, HCP: hydroxylamine reductase, IDH: isocitrate dehydrogenase, LSC: succinate–CoA ligase, MDH: malate dehydrogenase, NPD: ﻿nitronate monooxygenase, NiR: nitrite reductase, NiRA: nitrite reductase, NiRT: nitrite transporter, NIT: 2-oxoglutaramate amidase, NR: nitrate reductase, NRT: nitrate transporter, OTC: ornithine carbamoyltransferase, SDH: succinate dehydrogenase, unCPS: carbamoyl-phosphate synthase, URE: urease, UREG: urease accessory protein, URED/F: urease accessory protein, URT: urea transporter.

For *T. nordenskioeldii*, expression profiles of nitrogen metabolism-related genes were significantly different between the surface and SCM (Fig. 3a): high-affinity nitrate transporter (*NRT2*), nitrate reductase (*NR*), nitrite transporter (*NiRT*), nitrite reductase (*NiR*), urea transporter (*URT3*), ammonium transporter (*AMT6*), cyanate hydratase (*CYN*), urease accessory protein (*URED/F*), glutamate dehydrogenase (*GDH*), and aspartate aminotransferase (*GOT2*) were induced in surface samples; whereas ammonium transporter (*AMT2*), hydroxylamine reductase (*HCP*), and argininosuccinate synthase (*ASSY*) were induced in SCM samples. Two genes for nitrate transporters (*NRT1_2* and *NRT1_1*) were induced in surface and SCM samples, respectively. Among the induced genes in surface samples, *NRT1_1*, *NRT2*, *NR*, *NiRT*, and *NiR* contribute to an intake of nitrate from outside and inside of the cells and a reduction of nitrate to ammonia in the plastids^31^. Therefore, *T. nordenskioeldii* preferably uses extracellular and intracellular nitrate/nitrite as a nitrogen source for amino acids at the surface. *CYN* is involved in conversion of cyanate to NH_3_ in the cytoplasm^32^. Cyanate is a major nitrogen source for marine phytoplankton in oligotrophic regions of the mid Atlantic (up to 10% of total N uptake^33^). Widner *et al*.^33^ also showed heterogeneous distribution of cyanate throughout the water column and suggested release of cyanate to the outside of cells in SCM. In our analysis, *CYN* of *T. nordenskioeldii* was induced in the surface samples where nitrate concentration was low (Fig. 1d; Supplementary Table S1), suggesting that insufficient nitrogen was compensated by using cyanate at the surface. The induction of *CYN* was accompanied by high expression of *NR* in surface samples (Fig. 3a). This can be explained by consumption of intercellular cyanate, because cyanate is known to inhibit NR activity post-transcriptionally in *Chlorella vulgaris*^34^. *URT3* and *AMT6* were also induced in surface sample, contributing uptake of nitrogen sources, i.e. urea and ammonia, from the outside of cells. Urea hydrolysis for *T. nordenskioeldii* was uncertain because *URED* was induced but induction of other genes related to the urease complex was not observed as reported in Bender *et al*.^35^. On the other hand, among the induced genes in the SCM samples, an ammonia transporter, *AMT2*, was induced, while *AMT6* was induced in surface samples. AMT2 is located in plastid membranes and transports NH_4_^+^ from cytoplasm into plastids^36^. Thus, *T. nordenskioeldii* could activate the ammonium metabolism of plastids for use of intracellular ammonia as a nitrogen source for amino acids at the SCM during the matured spring bloom in this region. AMT6 is located in cytoplasmic membranes and transports NH_4_^+^ from outside of cells. The induction of *AMT6* is consistent with nitrogen limitation at the surface. Together with nitrogen metabolism, HCP and NR serve as electron sinks for redox balancing for hydroxylamine/NO and NO_2_^−^ reduction, respectively^37,38^. In our analyses, *HCP* and *NR* induction in SCM and surface samples, respectively, would explain diatoms’ control of intracellular redox state. Diatoms are unique among algal species with respect to possessing a complete urea cycle^31,39^. We detected gene expression for all enzymes of the urea cycle in surface and SCM samples; however, only *ASSY* was significantly induced in the SCM samples. ASSY catalyses the biosynthesis of L-argininosuccinate from aspartate and L-citrulline by the urea cycle^40^. These results suggest that the activity of the urea cycle might be regulated by the expression level of *ASSY*, closely related to TCA cycle activity. The aspartate is shared between the urea cycle and the TCA cycle because aspartate is reversibly synthesized from oxaloacetate by GOT2. The TCA cycle was active in surface samples because genes for TCA cycle enzymes were significantly induced: citrate synthase, isocitrate dehydrogenase, dihydrolipoamide succinyltransferase, succinyl-CoA synthetase, and fumarate hydrase. These results indicate that *T. nordenskioeldii* had increased mitochondrial respiration at the surface. The inactivation of urea cycle in the surface might prevent competition of oxaloacetate with TCA cycle. In the reaction of oxaloacetate biosynthesis by GOT2, the genes encoding GOT2 and GDH were both induced in surface samples. These suggest induction of 2-oxoglutarate production as a substrate for GOT2. In the SCM, nutrient concentration was high (Fig. 1d, Supplementary Table S1), and thus urea cycle might be activated to detoxify ammonium that was an intermediate metabolite derived from the nitrogen sources (e.g., nitrate). The activation of urea cycle can inactivate TCA cycle in the SCM.

The gene expression patterns of *C. debilis* were similar to those of *T. nordenskioeldii* in terms of nitrogen metabolism (Fig. 3b). For *C. debilis*, *AMTx*, *NiRT*, urea transporter (*DUR3*), and glutamine synthases (*GLNA* and *GLT*) were induced in surface samples. Interestingly, expression of *GOT2* had an opposite pattern compared to that for *T. nordenskioeldii*; *GOT2* of *C. debilis* and *T. nordenskioeldii* was induced in surface and SCM samples, respectively. Although reasons for this difference are unclear, *C. debilis* may regulate activity of urea cycle by transcriptional level of *GOT2* unlike *T. nordenskioeldii*.

### Factors for induction of *NRT2*, *NR*, and *AMT2*

**Figure 4:**
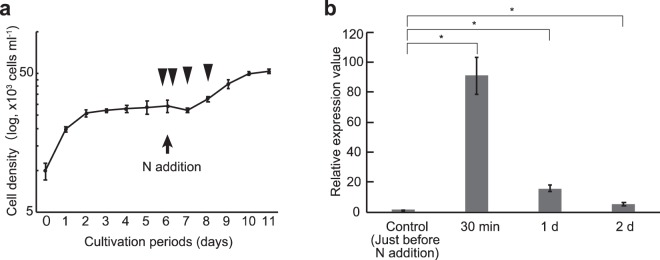
Culture experiments for induction of *NRT2* in *T. nordenskioeldii*. Transcriptional differences of *NRT2* in *T. nordenskioeldii* before/after nitrogen addition under nitrogen-depleted conditions. (**a**) Growth curve of the *T. nordenskioeldii* strain. The strain was cultivated for 6 days, then both NaNO_3_ and NH_4_Cl were added (arrow). The cells were sampled four times, just before the nitrogen addition, and after 30 min, 1 day, and 2 days (arrowheads). Error bars represent standard deviation (SD). (**b**) Relative expression values of *NRT2* in *T. nordenskioeldii*, normalized to the expression value of 18S rRNA. Error bars represent SD. P-values are shown on bars (t-test). Asterisks represent significantly different expression values (p < 0.01).

**Figure 5:**
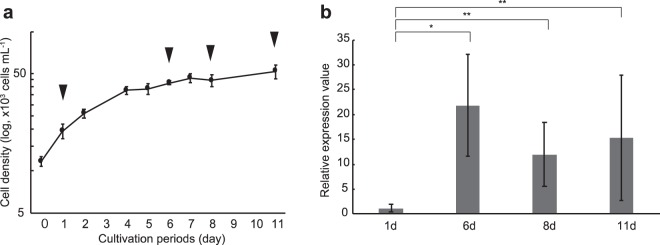
Culture experiments for induction of *AMT2* in *T. nordenskioeldii*. Transcriptional differences of *AMT2* in *T. nordenskioeldii* under nitrogen-repleted conditions. (**a**) Growth curve of the *T. nordenskioeldii* strain. The strain was cultivated for 11 days, and sampled four times, 1, 6, 8, and 11 days after starting cultivation (arrowheads). The sampled cells were washed, transferred into new medium (i.e., nitrogen-repleted 1/10 N K medium), and incubated for 2 h in the dark, and the RNA was extracted. Error bars represent SD. (**b**) Relative expression values of *AMT2* in *T. nordenskioeldii*, normalized to the expression value of 18S rRNA. Error bars represent SD. P-values are shown on bars (t-test). Asterisks represent significantly different expression values (p < 0.01). Double asterisks represent non-significant expression values (p ≥ 0.01).

To elucidate factors causing the differential gene expression in nitrogen metabolism, we conducted qPCR analysis of *T. nordenskioeldii*, cultured in a combination of nitrogen forms (i.e., NH_4_^+^ and NO_3_^−^) under various conditions described in Supporting Text and Supplementary Fig. S8. In particular, we focused on *NRT2*, *NR*, and *AMT2*, which were highly expressed in the surface and SCM samples, respectively, in *T. nordenskioeldii* (Fig. 3a). After adding nitrogen in the form of both NH_4_^+^ and NO_3_^−^ to nitrogen-depleted *T. nordenskioeldii*, the cells began to grow and kept growing for 4 days (Fig. 4a). The relative expression values of *NRT2* increased to 90-fold within 30 min after nitrogen addition (p < 0.01, t-test), and then decreased in the next 1 and 2 days (Fig. 4b). The significant increase in the *NRT2* transcript was also seen when only NO_3_^−^ was added (Supplementary Fig. S8c). *NR* expression was also induced by the NO_3_^−^ addition (Supplementary Fig. S8c). On the other hand, *AMT2* expression was not significantly different (p > 0.05) (Supplementary Fig. S8c–e). These results indicate that *NRT2* in *T. nordenskioeldii* is induced by nitrate, as shown in an alga and plants^41–44^. Next, we investigated relationships between population growing state and gene expression by comparing *AMT2* gene expression in the culture strains (Fig. 5a,b). The *AMT2* expression values in nutrient-replete culture increased to 22-fold in 6 days’ culturing (p < 0.01) and the level was maintained toward the stationary phase (8 and 11 days’ culturing) (Fig. 5b). Meanwhile, NH_4_^+^ addition did not affect *AMT2* expression (Supplementary Fig. S8d,e). Therefore, *T. nordenskioeldii AMT2* was induced during the actively growing phase, particularly late log phase, in the presence of NH_4_^+^ outside of the cell. Our results indicate that diatoms preferably use nitrate, but temporally use ammonium in the actively growing phase during the bloom-maturing period in their natural environment. Under active cell growth, the accessible but toxic ammonium in the cells would be immediately converted into detoxified forms, thus ammonium is less available.

Low light intensity also induced expression of *NRT2* (Supplementary Fig. S8b). The relative expression values of *NRT2* under low light (~80 µ photons m^−2^ s^−1^) increased to three-fold compared to high light (~260 µ photons m^−2^ s^−1^). However, the induction of *NRT* by nitrate addition is more efficient than that by low light intensity. These results indicate that the supply of nitrate can be a main factor to regulate *NRT2* expression.

The results of the culturing experiments are consistent with the cellular response of major diatoms in nature. Our field study showed that the NO_3_^−^ concentration at the surface was low but sharply increased below ~20 m depth (Fig. 1d; Supplementary Table S1), implying that the surface is nitrogen limited. Additionally, small amount of nitrate might be supplied by turbulence. This situation can explain high expression of genes for nitrate uptake in both *T. nordenskioeldii* and *C. debilis* in surface samples as described above for *NRT2* of *T. nordenskioeldii*. Similar situation that diatoms had quick response to upwelled nitrate was reported^13^. Diatom cells at the SCM is under initial decline phase of the bloom, probably corresponding to late log phase in the culturing experiments, which can explain the induction of *AMT2* in SCM sample. The transcriptional induction of *NRT2*, *NR*, and *AMT2* can be used as a direct index of development stages of diatom blooms.

### Unique transcriptional response of genes for iron transporters in *Chaetoceros debilis*

**Figure 6:**
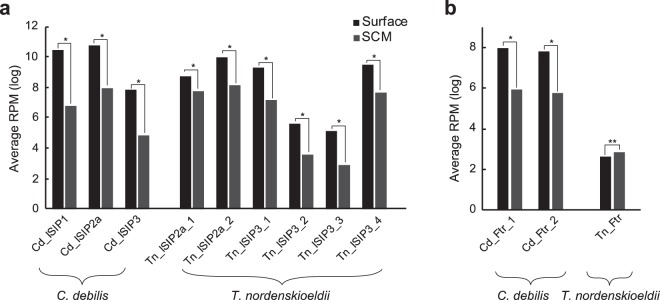
Changes in expression values of genes for sensing and intake of iron. (**a**) Average reads per million (RPM) of genes for iron sensing (*ISIP1* and *ISIP3*) and iron accumulation (*ISIP2a*) in *C. debilis* and *T. nordenskioeldii*. (**b**) Average RPM of genes for iron intake (*Ftr*) in *C. debilis* and *T. nordenskioeldii*. FDRs are shown above bars. Asterisks represent significantly different RPM (FDR < 0.01). Double asterisks represent non-significant RPM (FDR ≥ 0.01).

A mesoscale iron-enrichment experiment in the western subarctic North Pacific (neighbouring region for our experiment) showed that the dominant diatom species changed from *Pseudo-nitzschia turgidula* to *C. debilis* after iron addition^14^. During the experiment, *C. debilis* was more sensitive to iron concentration change than the other diatoms in this region, and had a high growth rate, 2.6 doublings per day, after iron addition. Concentration of dissolved iron initially reached 2.9 nM in a patch and subsequently rapidly decreased. In our analyses, both *C. debilis* and *T. nordenskioeldii* had high expression of *ISIP3*, encoding an iron starvation-induced protein, at the surface (Fig. 6a). *C. debilis* also induced another gene for ISIP (*ISIP1*). ISIP1 and ISIP3 are putative receptors and co-receptors, respectively, which are localized on the cell membrane, and induced in low iron environments^45^. Additionally, *T. nordenskioeldii* and *C. debilis* had high expression of *ISIP2a*, which concentrates iron at the cell surface^46^, in the surface layer (Fig. 6a). Therefore, *T. nordenskioeldii* and *C. debilis* are iron-depleted in iron-limited environments such as the surface of the subarctic Pacific^47^. Interestingly, another iron-related gene, iron permease (*Ftr*), showed a different expression pattern between *T. nordenskioeldii* and *C. debilis* (Fig. 6b): *C. debilis* had significantly higher expression of *Ftr* at the surface than SCM, whereas *T. nordenskioeldii* did not have significantly different expression. These results suggest that *C. debilis*, but not *T. nordenskioeldii*, transcriptionally regulates iron intake systems during spring diatom bloom in this region. Moreover, those results could also explain the high sensitivity of *C. debilis* to the temporal increase in iron concentration in the mesoscale iron-enrichment experiments. *C. debilis* can rapidly grow corresponding to the iron addition because *C. debilis* produces more iron intake systems than the other diatoms (e.g., *T. nordenskioeldii*) in the surface layer.

### Suspending strategy at the SCM

Changing the buoyancy is one of the main strategies of diatoms to adapt to marine environments^48^. In the SCM, genes for phospholipid:diacylglycerol acyltransferase (PDAT), which is involved in triacylglycerol synthesis from phospholipid^49^, were induced in *T. nordenskioeldii* and *C. debilis* (Supplementary Fig. S9). Lipid accumulation is responsible for the floating strategy of marine and freshwater algae ^50–52^. However, the floating strategies are not simple for diatoms; indeed lipid content of a freshwater diatom *Asterionella* is not enough to make it float^53^. Body shape, altered, for example, by chitin fibres, can also affect buoyancy^51,54^. Our data showing high expression of *T. nordenskioeldii* chitin synthase genes in surface samples indicate production of chitin fibres (Supplementary Fig. S10a), which would form long fibres on the cell membrane^55,56^. Thus, *T. nordenskioeldii* might differentially express genes to regulate their buoyancy to adapt to local environments (i.e., surface or SCM) within a spring diatom bloom in the Oyashio region. Interestingly, the gene for plastid ftsZ, which is a plastid division protein^57,58^, was induced at the surface (Supplementary Fig. S10b), suggesting frequent plastid division at the surface, which may correspond to light intensity. In contrast, the gene of *C. debilis* was not induced because *C. debilis* has a single plastid per cell^59^.

## Conclusions

Here, we performed metatranscriptome analyses of the bloom-forming diatoms, focusing differences between two depths, and cultivation experiments to reproduce the expression patterns of genes for nitrogen intake. We reveal the transcriptional complexity of diatom bloom formation. It is thought that suspension and sinking are important for diatoms to grow in a suitable environment, and we indicate that the vertical relocation should affect cell responses of diatoms, and trigger bloom formation. Expression of *AMT2* is likely to be closely related to the bloom-maturing, and thus it can be used for quantitative monitoring of diatom bloom formation. Combining metatranscriptome and strain-based analysis using isolated cultures enables elucidation of population dynamics and cellular responses in various types of algal blooms, such as raphidophyte bloom, cyanobacterial bloom, and *Aureococcus* bloom.

## Materials and Methods

### Seawater sampling and prediction of development stages of the diatom bloom

Seawater sampling was conducted in the Oyashio region of the western North Pacific (Station A4: 42.25°N, 145.13°E) ~2 h after sunset on May 16, 2016, onboard the R/V *Wakataka Maru*. Seawater samples were collected using the Niskin bottles. 110 L of seawater samples for metatranscriptome were collected at 10 m depth within the surface mixing layer (10 m depth) and at 30 m depth of SCM layer after sunset. Each sample was pre-filtered using a plankton net (100 µm mesh size) and the cells >20 µm were concentrated to 45 mL using a plankton net (20 µm mesh size), then filtered onto a 0.2 µm pore size polycarbonate Nuclepore filter (Whatman, Dassel, Germany) with gentle vacuum pressure (<150 mmHg). The sampling, concentrating, and filtering the cells for metatranscriptome analysis was completed within 15 min. The filters were immersed in RNAlater (Ambion, Austin, TX, USA) for 15 min at room temperature, and flash frozen in liquid nitrogen, then stored at −80 °C until analysis. Samples for nutrients and chlorophyll *a* concentration analysis were collected from nine layers (0, 10, 20, 30, 40, 50, 60, 80, and 100 m depth). Subsamples of 1 L were immediately fixed with acid Lugol’s solution (4% final concentration) and preserved at 4 °C. Vertical profiles of temperature and salinity were measured using a conductivity-temperature-depth profiler. To elucidate development stages of the diatom bloom, an index for the development of spring diatom blooms (*I*_devel_)^20^ was used. For this index, diatom blooms are divided into three stages: pre-bloom (*I*_devel_ < 0.15), growth phase of the bloom (*I*_devel_ = 0.15 to 0.50), and decline phase of the bloom (*I*_devel_ > 0.50).

### Identification of major diatom group

For enumeration of phytoplankton cells, fixed samples were concentrated by reverse filtration through 2 µm nucleopore filters. In the concentrated samples, diatom species were identified following Hasle and Syvertsen^60^, and their abundance (cells L^−1^) was estimated using inverted light microscopy.

### RNA extraction, quality control, and sequencing

RNA was extracted from three independent samples per depth using RNeasy Mini Kit (Qiagen Ltd., Venlo, The Netherlands), with slight modifications. The diatoms on filters with RNAlater were incubated with 700 µL of buffer RLT and 7 µL of 2-mercaptoethanol on ice. Cells were collapsed using a Mini Bead Beater (Cole-Parmer, Vernon Hills, IL, USA) with glass beads (0.05 g of 0.1 mm beads and 0.05 g of 0.5 mm beads, Bertin, Rockville, MD, USA) at 5,000 rpm for 50 s, and the filters were removed after centrifugation at 15,000 rpm for 3 min. Subsequently, the manufacturer’s protocol was followed. Genomic DNA was removed using the TURBO DNA-free Kit (Thermo Fisher Scientific, Waltham, MA, USA), and the quality of the RNA was checked using TapeStation (Agilent Technologies, Santa Clara, USA). Genomic DNA was checked by using PCR and details were described in Supporting Information and Supplementary Fig. S11. RNA libraries were constructed using NEBNext Ultra Directional RNA Library Prep Kit for Illumina (New England BioLabs, Ipswich, MA, USA) according to the manufacturer’s protocol. Sequencing was performed using the MiSeq System (Illumina Inc., San Diego, CA, USA) with MiSeq Reagent Kits v2 (250 bp × 2) or v3 (300 bp × 2) (Illumina) (Supplementary Table S4). In total, 42,073,468 paired-reads (8,390,580,790 bp) were acquired, with more than 4.9 million paired-reads (1.0 Gbp) per sample.

For reference, RNA-seq analysis of *T. nordenskioeldii* NIES-4227, which was isolated from the Oyashio region of the western North Pacific, was performed. The cells were cultivated in 100 mL of L1 medium^61^ for 2 days under white LED light (~20 µmol photons m^−2^ s^−1^) with 14 h:10 h light:dark cycles. Cells were collected by gentle centrifugation during the light and dark phases, and equal amounts of the light and dark RNA samples were mixed. RNA was extracted using the RNeasy Mini Kit (Qiagen) with slight modifications. The cells were incubated with 350 µL of buffer RLT and 3.5 µL of 2-mercaptoethanol on ice, and vortexed to mix. The cells were collapsed using a Mini Bead Beater (Cole-Parmer) with glass beads (0.05 g of 0.5 mm beads, Bertin) at 5,000 rpm for 30 s. Subsequently, the manufacturer’s protocol was followed. DNase treatment and RNA library construction were performed as described above. Sequencing was performed using the MiSeq System (Illumina) with MiSeq Reagent Kits v3 (300 bp × 2) (Illumina). In total, 13,440,632 paired-reads (2,939,690,202 bp) were obtained. All of the reads were trimmed using Trimmomatic 0.36 with default options^62^. The RNA-seq reads of the metatranscriptome were deposited in GenBank/ENA/DDBJ with accession numbers DRA006756, DRA008069, and DRA008900. The RNA-seq reads of *T. nordenskioeldii* were deposited in GenBank/ENA/DDBJ with accession number DRA006754.

### Assembly of RNA-seq reads for reference of genes

Four major species of the diatom bloom were selected: *T. nordenskioeldii*, *C. socialis*, *C. debilis*, and *F. cylindrus*. For references, transcripts data of *F. cylindrus* were downloaded from JGI website (https://genome.jgi.doe.gov/Fracy1/Fracy1.home.html)^63^, and RNA-seq data of *C. socialis* and *C. debilis* were downloaded from the NCBI SRA database (accession numbers SRR1205835 and SRR1296919, respectively). RNA-seq of *T. nordenskioeldii* was obtained in this study as described above. All of the RNA-seq reads were assembled using Trinity v 2.2.0^64^. Coverages were calculated by RSEM^65^ and bowtie2^66^. Isoforms with the highest coverages were extracted using a script in Trinity package as reference genes. Finally, 34,797, 69,789, and 28,069 transcripts for *T. nordenskioeldii*, *C. socialis*, and *C. debilis*, respectively, were acquired.

### Mapping and DEG analyses

RNA-seq reads from the environment samples were mapped to the reference genes using segemehl version 0.3.2^67^ with similarity >85%. The hit strategy was best-hit only, which aligns only the most similar read to the reference under similarity criteria. Read count data were split into respective species and used for differentially expressed gene (DEG) analyses. Genes without any mapped reads were removed from the dataset. Read count normalization and DEG analyses were performed with likelihood ratio tests using edgeR 3.18.1^68^, and DEGs were defined with false discovery rate (FDR) < 0.01.

### Functional analyses

Functional analyses of DEGs were performed using Kyoto Encyclopedia of Genes and Genomes (KEGG)^69^ and eggNOG mapper 1.0.3^70,71^. Genes for nitrogen metabolism were also identified by performing blastp with proteins of *T. pseudonana*^31^.

### Cultivation experiments for variable gene expression of *AMT2*, *NR* and *NRT2*

To find factors influencing gene expression of *AMT2*, *NR*, *and NRT2*, *T. nordenskioeldii* NIES-4227 cells were cultivated under various conditions and subjected to qPCR analyses. All cultivations were performed using filter-sterilized 1/10 N K medium or 1/100 N K medium with artificial sea water (Wako, Osaka, Japan or Red Sea, Huston, TX, USA). These are modified K media^72,73^ with 1/10 or 1/100 concentrations of nitrogen source (original concentrations in K medium: 882 µM NaNO_3_ and 50 µM NH_4_Cl, respectively). Cell numbers were counted manually using a 4 Grid Cell Counter Plate (Fuchs Rosental model, Watoson Co., Ltd., Tokyo, Japan). Detailed methods for the cultivation and qPCR analyses are described in Supporting Text. All of the cultivation and qPCR analyses had three independent technical and biological replicates.

## Supplementary information

Supporting text

Supplementary Tables
