# Supplementary material for: Depth-dependent transcriptomic response of diatoms during spring bloom in the western subarctic Pacific Ocean
Source: Sci Rep. 2019 Oct 10;9:14559. doi: 10.1038/s41598-019-51150-8 (PMC6787086; doi:10.1038/s41598-019-51150-8)
Supplement: Supplementary file 1 — Supporting text [file 41598_2019_51150_MOESM1_ESM.docx]

**Depth-dependent transcriptomic response of diatoms during spring bloom in the western subarctic Pacific Ocean**.

Shigekatsu Suzuki^1*^, Takafumi Kataoka^2^, Tsuyoshi Watanabe^3^, Haruyo Yamaguchi^1^, Akira Kuwata^3^, and Masanobu Kawachi^1^

^1^ Center for Biology and Ecosystem Studies, National Institute for Environmental Studies, 16-2 Onogawa, Tsukuba, Ibaraki, Japan

^2^ Faculty of Marine Science and Technology, Fukui Prefectural University, 1-1 Gakuen-cho, Obama, Fukui, Japan

^3^ Tohoku National Fisheries Research Institute, Fisheries Research Agency, 3-27-5 Shinhama-cho, Shiogama, Miyagi, Japan

*Correspondence: S Suzuki, National Institute for Environmental Studies, 16-2 Onogawa, Tsukuba, Ibaraki, 305-0053, Japan, Phone: +81 29 850 2204, Fax: +81 29 850 2587, e-mail: suzuki.shigekatsu@nies.go.jp

**Supporting text**

*Taxonomic analyses of RNA-seq reads*

Taxonomic analyses of RNA-seq reads of the environmental samples were performed. Ten percent of all of the reads were randomly subsampled, and homology-searched using DIAMOND v0.9.10.111^2^ with default options against the NCBI non-redundant database. Taxonomic paths were depicted using Megan Community Edition version 6.9.0^3^ (Supplementary Fig. S1). The result shows that diatoms were the most transcriptionally active group in eukaryotes in this field.

*Growth rates of* T. nordenskioeldii *in low nitrate concentration*

*Thalassiosira nordenskioeldii* NIES-4227 was pre-cultured in 25 mL nitrogen-free K medium^4,5^ for two days under white LED light (~20 µmol photons m^-2^ s^-1^) with 14 h:10 h light:dark cycles. After washing cells once, cells were cultivated in 4 mL modified ammonium-free K media with 0.88 µM or 8.8 µM NaNO_3_, which corresponded to the concentration of the surface (0.75 µM) and SCM (10.08 µM) samples, under white LED light (~50 µmol photons m^-2^ s^-1^). Cell numbers were counted manually using a 4 Grid Cell Counter Plate (Fuchs Rosental model, Watoson Co., Ltd., Tokyo, Japan). Initial cell density was 48,125 (0.88 µM) and 60,313 cells mL^-1^ (8.8 µM). Growth rates were calculated using the cell densities for the initial three days, and *T. nordenskioeldii* showed similar growth rate (0.88 µM: µ = 0.21±0.05 d^-1^, 8.8 µM: 0.15±0.03 d^-1^) under these two nitrogen conditions (Supplementary Fig. S2).

*Cloning analyses and phylogenetic analyses for spring blooms sampled in the cruise in 2015*

DNA-base TA-cloning analysis for the 18S rRNA gene was conducted. Genomic DNA was extracted from cells sampled from spring bloom at Station A4 in 2015 using DNeasy Plant Mini Kit (Qiagen), and the 18S rRNA gene was PCR amplified using the eukaryote-specific primer set of SR1 and SR12^6^. The amplicon was then inserted into a plasmid (pGEM-T Easy Vector, Promega, Madison, WI, USA) and isolated by picking up an *Escherichia coli* (strain DH5α) colony containing the transformed plasmid. The inserted 18S rRNA gene was screened by examining the NCBI non-redundant database for partial sequence obtained using a M13 forward primer, then the full sequence of the 18S rRNA was obtained (3730 DNA analyzer, Applied Biosystems, Foster City, CA, USA). A phylogenetic tree was constructed using 1,680 nucleotides of 28 diatoms including the 11 sequences obtained by TA-cloning analysis (Supplementary Fig. S4). The sequences were aligned using Mafft v7.305b^7^, and manually curated on MEGA 6^8^. Model test and maximum Likelihood (ML) analyses were performed using IQ-tree multicore-version 1.41^9^ with the TIM2 + I + G4 substitution model. The non-parametric bootstrapping was replicated 100 times. The 18S rRNA sequences obtained were deposited in GenBank/ENA/DDBJ with accession number LC371031-LC371044.

*Cultivation experiments and qPCR for* AMT2*,* NR*,* *and* NRT2

To find factors influencing gene expression of *AMT2*, *NR*, *and NRT2*, *T. nordenskioeldii* NIES-4227 cells were cultivated under various conditions as described below and subjected to qPCR analyses. All cultivations were performed using filter-sterilized 1/10 N K medium or 1/100 N K medium with artificial sea water (Wako, Osaka, Japan or Red Sea, Huston, TX, USA). These are modified K media^4,5^ with 1/10 or 1/100 concentrations of nitrogen source (original concentrations in K medium: 882 µM NaNO_3_ and 50 µM NH_4_Cl, respectively). Cell numbers were counted manually using a 4 Grid Cell Counter Plate (Fuchs Rosental model, Watoson Co., Ltd). Cells were rapidly collected by gentle centrifugation, avoiding strong light stimulation, and then incubated with 1 mL of pre-cooled Trizol reagent (Invitrogen). RNA was extracted according to the manufacturer’s protocol. DNase treatment was performed as described above. cDNA was synthesized using ReverTra Ace qPCR RT Kit (Toyobo Co., Ltd., Osaka, Japan). qPCR was performed using SYBR Premix Ex Taq II (Tli RNaseH Plus) (Takara, Shiga, Japan) and the Thermal Cycler Dice Real Time System (Takara). Primers for the qPCR were designed in this study for the following *T. nordenskioeldii* transcripts: 18S rRNA, ammonium transporter 2 (*AMT2*), nitrate reductase, and high-affinity nitrate transporter (*NRT2*). The sequences were as follows: 18SrRNA-F: 5’-TCAAACACGGGAAAACTTACCA-3’, 18SrRNA-R: 5’-AACCAGACAAATCACTCCACCA-3’, AMT2-F: 5’-TGTTGAGAAGCCAGCGATTT-3’, AMT2-R: 5’-CGCTATCCCCGTTCACTTTT-3’, NR-F: 5’-GCCAACCAAATCCGCAACATTG-3’, NR-R: 5’- TCACGCGTGTTGAAATCACCAC-3’, NRT-F: 5’-CATCATCATCAACCTTCTTCTCAAC-3’, and NRT-R: 3’-GGCACCTTCCACCACTCTTC-5’. Relative expression values were calculated using the ∆∆CT method, and normalized using 18S rRNA. All of the cultivation and qPCR analyses had three independent technical and biological replicates.

*Cultivation experiments for variable gene expression of* AMT2 *and* NRT2*.*

*Relationships between temperature variation and gene expression.*

The cells were cultivated for 2 days at 10°C in 20 mL of 1/10 N K medium under L:D = 12 h:12 h white LED light conditions (~80 µ photons m^-2^ s^-1^). Initial cell concentration was ~0.8 × 10^4^ cells mL^-1^. Then, the cells were incubated for 2 h in the dark. The control cells were collected, whereas the other cells were transferred to 5°C in a water bath, incubated for 30 min, and then collected. The relative expression values of *NRT2* and *AMT2* were not significantly different between 10°C and 5°C (p ≥ 0.01) (Supplementary Fig. S8a).

*Relationships between light intensity and gene expression.*

The cells were cultivated for 2 days at 5°C in 20 mL of 1/10 N K medium under L:D = 12 h:12 h white LED light conditions. Initial cell concentration was ~1.9 × 10^4^ cells mL^-1^. The light intensities were ~80 (low light) or ~260 (high light) µ photons m^-2^ s^-1^. After 2 days’ cultivation, the cells were incubated for 2 h in the dark, and rapidly collected. The relative expression values of *NRT2* were slightly different (p < 0.01), and the cells under high light had lower gene expression than those under low light (Supplementary Fig. S8b). The relative expression values of *NR* and *AMT2* were not significantly different (p ≥ 0.01). These results indicate that expression of *NRT2* is down-regulated by strong light, whereas that of *NR* and *AMT2* is not affected.

*Relationships between nitrate or ammonium addition, and the gene expression under nitrogen-depleted conditions.*

The cells were cultivated for 2 days at 5°C in 20 mL of 1/100 N K medium (i.e., nitrogen-depleted medium including 8.8 µM NaNO_3_ and 0.5 µM NH_4_Cl) under L:D = 12 h:12 h white LED light conditions (~80 µ photons m^-2^ s^-1^). Initial cell concentration was ~0.5 × 10^4^ cells mL^-1^. After 2 days’ cultivation, the cells were incubated for 2 h in the dark. The control cells were collected, whereas the other cells were provided with NaNO_3_ (final concentration: ~88.2 µM) or NH_4_Cl (final concentration: ~5.0 µM), incubated for 30 min in the dark, and then collected. In NaNO_3_-treated samples, the relative expression values of *NRT2* and *NR* were significantly high (p < 0.01), and >35 and >7 times higher than those of the control, respectively (Supplementary Fig. S8c). Expression values of *AMT2* were not significantly different after NH_4_Cl addition (Supplementary Fig. S8d). These results indicate that expression of *NRT2* and *NR* is induced immediately by NO_3_^-^ addition under nitrogen depletion.

*Relationships between both nitrate and ammonium addition, and gene expression under nitrogen-depleted conditions.*

This experiment is also described in Figure 3 in the main text. The cells were cultivated for 6 days at 5°C in 25 mL of 1/100 N K medium under L:D = 12 h:12 h white LED light conditions (~80 µ photons m^-2^ s^-1^). Initial cell concentration was ~1.0 × 10^4^ cells mL^-1^. After 6 days’ cultivation, the cells were incubated for 2 h in the dark. The control cells were collected, whereas the other cells were provided with NaNO_3_ (final concentration: ~88.2 µM) and NH_4_Cl (final concentration: ~5.0 µM) and cultivated for an additional 30 min, 1 day, and 2 days, and then each sample was collected 2 h after the start of the dark phase. The cells after 6 days’ cultivation were nitrogen-depleted because the cells in stationary phase started to grow again after nitrogen addition (Fig. 4a, b). The expression values of *NRT2* are described in the main text (Fig. 4b). Those of *AMT2* were not significantly different (Supplemental Fig. S8e).

*Relationships between growth state and gene expression under nitrogen repletion.*

This experiment is also described in Figure 5 in the main text. The cells were cultivated for 10 days at 5°C in 30 mL of 1/100 N K medium under L:D = 12 h:12 h white LED light conditions (~80 µ photons m^-2^ s^-1^). Initial cell concentration was ~1.0 × 10^4^ cells mL^-1^. After 2’, 6’, 8’, and 10 days’ cultivation (Fig. 5a), 10 or 5 mL of cells were sampled at the start of the dark phase. Each sample was collected by gentle centrifugation and washed using 1/10 K medium. Then, the cells were incubated in 5 mL of 1/10 N K medium for 2 h in the dark and collected. The expression values of *NRT2* are described in the main text (Fig. 5b). Those of *NRT2* after 6 days of cultivation were significantly different from after 1 day of cultivation (p < 0.01), and >100 times higher (Supplementary Fig. S8f). The values continued to increase after 8 days’ and 11 days’ cultivation. These results were caused by nitrogen depletion of the cells.

*Confirmation of removal of DNA contamination.*

After the DNase treatment, DNA contamination in the RNA samples was checked by reverse transcription PCR (RT-PCR). cDNA was synthesized using ReverTra Ace -α- (Toyobo) with random primers and ~30–190 ng of the RNA samples. 18S rRNA genes were amplified by PCR using TaKaRa Ex Taq (Takara) with universal primers, 63f and 1818r^1^. The PCR was performed using the following conditions: initial denaturation at 96°C for 2 min; 30 cycles of 15 sec at 96°C, 15 sec at 55°C and 1.5 min at 72°C; and final extension at 72°C for 5 min. 18S rRNA genes were amplified using each cDNA sample; however, no product was amplified using each RNA sample (Supplementary Fig. S11a, b). These results show that DNA contamination in the RNA samples has been digested by our DNase treatment.

**Supplementary references**

1. Lepere, C. *et al.* Whole-genome amplification (WGA) of marine photosynthetic eukaryote populations. *FEMS Microbiol. Ecol.* **76**, 513–523 (2011).

2. Buchfink, B., Xie, C. & Huson, D. H. Fast and sensitive protein alignment using DIAMOND. *Nat. Methods* **12**, 59–60 (2014).

3. Huson, D. H. *et al.* MEGAN Community Edition - interactive exploration and analysis of large-scale microbiome sequencing data. *PLoS Comput. Biol.* **12**, e1004957 (2016).

4. Keller, M. D., Seluin, R. C., Claus, W. & Guillard, R. R. L. Media for the culture of oceanic ultraphytoplankton. *Journal of Phycology* **23**, 633–638 (1987).

5. Keller, M. D. & Guillard, R. R. L. Factors significant to marine diatom culture. in *Toxic Dinoflagellates* (eds. Anderson, D. M., White, A. W. & Baden, D. G.) 113–116 (Elsevier, 1985).

6. Nakayama, T. *et al.* The basal position of scaly green flagellates among the green algae (Chlorophyta) is revealed by analyses of nuclear-encoded SSU rRNA sequences. *Protist* **149**, 367–380 (1998).

7. Katoh, K. & Toh, H. Recent developments in the MAFFT multiple sequence alignment program. *Brief. Bioinform.* **9**, 286–98 (2008).

8. Tamura, K., Stecher, G., Peterson, D., Filipski, A. & Kumar, S. MEGA6: molecular evolutionary genetics analysis version 6.0. *Mol. Biol. Evol.* **30**, 2725–2729 (2013).

9. Nguyen, L.-T., Schmidt, H. A., von Haeseler, A. & Minh, B. Q. IQ-TREE: a fast and effective stochastic algorithm for estimating maximum-likelihood phylogenies. *Mol. Biol. Evol.* **32**, 268–274 (2015).

**Supplementary Figure S1. Proportion of taxonomic identification of RNA-seq reads**

Proportion of taxonomic identification of RNA-seq reads is shown. The RNA-seq reads were obtained from environmental metatranscriptome samples. The reads were identified as diatoms, Alveolata, Haptophyta, Opisthokonta, other stramenopiles, Viridiplantae, and others. Reads without DIAMOND-hits were not considered.

**Supplementary Figure S2. Growth of *T. nordenskioeldii* in low nitrate concentration.**

Growth curves of *T. nordenskioeldii* in low nitrate concentration are shown. Initial nitrate concentration is 0.88 µM and 8.8 µM. Growth rates are calculated using the cell densities for the initial three days.


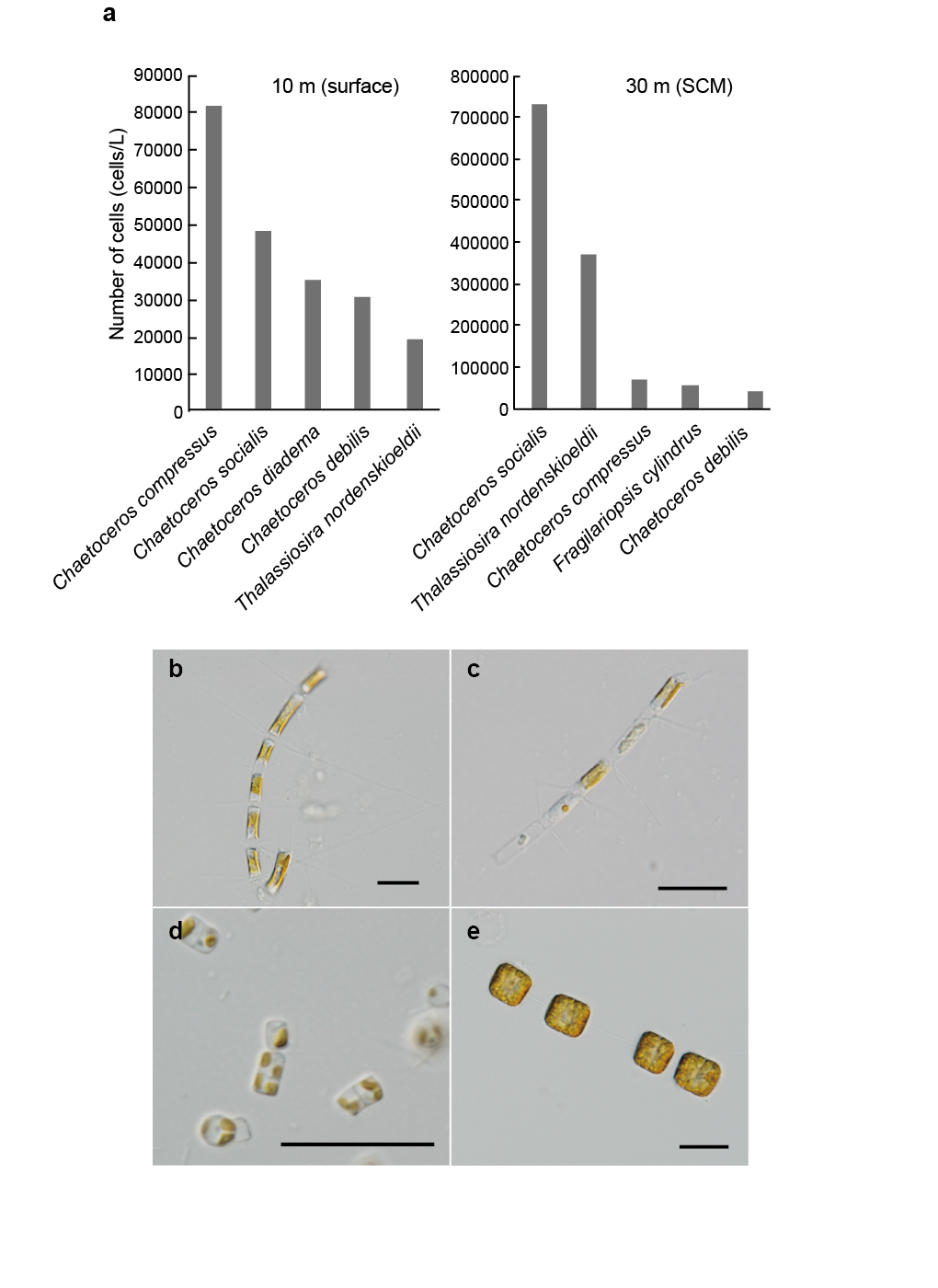


**Supplementary Figure S3. Direct cell counting of diatom species in the spring bloom and images of diatom species used in this study**

(**a**) Numbers of diatom cells mainly composed of the bloom in the surface and SCM samples. The cell numbers were manually counted. Only the top five species are shown. (**b–e**) Representative images of diatom species used in this study. Scale bars represent 20 µm. (**b**) *C. socialis* (NIES-377), (**c**) *C. debilis* (NIES-3710), (**d**) *F. cylindrus* (NIES-3966), and (**e**) *T. nordenskioeldii* (NIES-4227).

**Supplementary Figure S4. Phylogenetic analysis using 18S rRNA genes of diatoms**

ML tree of diatoms using 18S rRNA sequences. Red-colored operation taxonomic units represent sequences obtained from our cloning analyses of the diatom bloom in 2015. Bold lines show BP = 100.

**Supplementary Figure S5. Mapping efficiency of four diatom species**

Average proportions of reference genes with mapped reads are shown. Error bars represent standard deviation (SD) of triplicates.


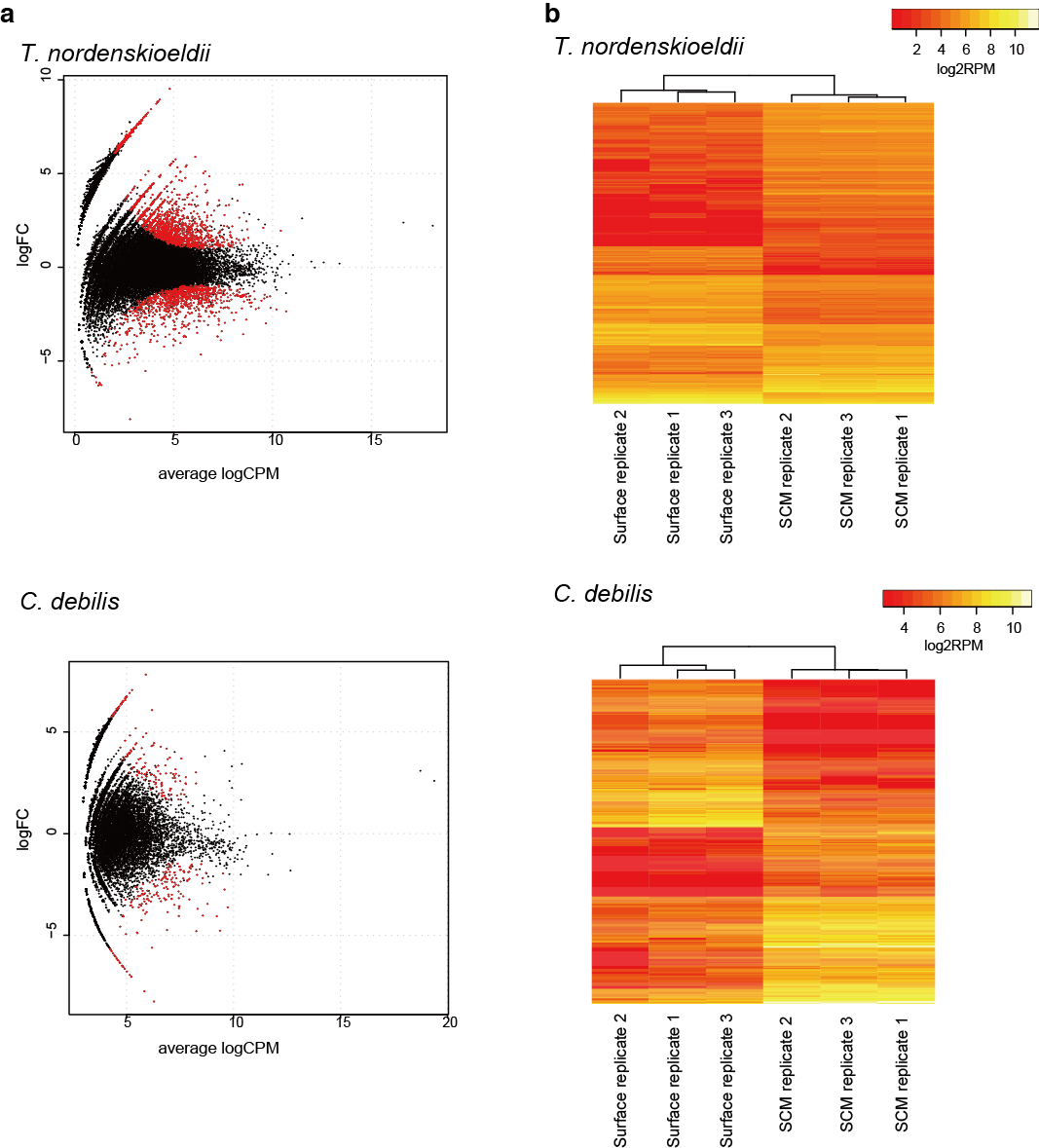


**Supplementary Figure S6. Differentially expressed gene (DEG) analyses of *T. nordenskioeldii* and *C. debilis*.**

(a) MA plot of the analyses. Genes with FDR < 0.01 are shown as red plots. (b) Clustering analyses of the logarithmic RPM (read count per million reads)values. Up-regulated and down-regulated genes are shown in yellow and red, respectively.

**Supplementary Figure S7. Changes in expression values of genes for silicon transporters (*SIT1*)**

Average logarithmic reads per million (RPM) of genes for silicon transporters (*SIT1*) and (b) heat shock family transcription factors. Differently expressed genes are only shown (FDR < 0.01).

**Supplementary Figure S8. Culture experiments under various conditions.**

Relative expression values of *NRT2* and *AMT2* in *T. nordenskioeldii*, normalized to the expression value of 18S rRNA. Error bars represent SD. P-values are shown on bars (t-test). (a) Relative gene expression values under temperature variation from 10°C to 5°C. (b) Relative gene expression values under different light intensities (~80 and ~260 μ photons/m^2^/s). (c, d) Relative gene expression values after nitrate (c) or ammonia (d) addition in a nitrogen-deficient medium. (e) Relative gene expression values after addition of both nitrate and ammonia under nitrogen depletion. (f) Relative gene expression values in the presence of nitrogen resources.

**Supplementary Figure S9. Putative metabolism pathway of *T. nordenskioeldii* and *C. debilis* for lipid biosynthesis.**

Metabolites and enzymes of *T. nordenskioeldii* (left) and *C. debilis* (right) are shown. The names of enzymes (circles) are shown in bold type. Genes induced in surface and SCM samples are depicted as red- and blue-colored circles, respectively.

**Supplementary Figure S10. Changes in expression values of genes for chitin synthases and platid FtsZ of *T. nordenskioeldii***

(a) Average logarithmic reads per million (RPM) of genes for chitin synthases and (b) plastid FtsZ of *T. nordenskioeldii*. Differently expressed genes are only shown (FDR < 0.01). For *C. debilis*, these genes are not differently expressed.


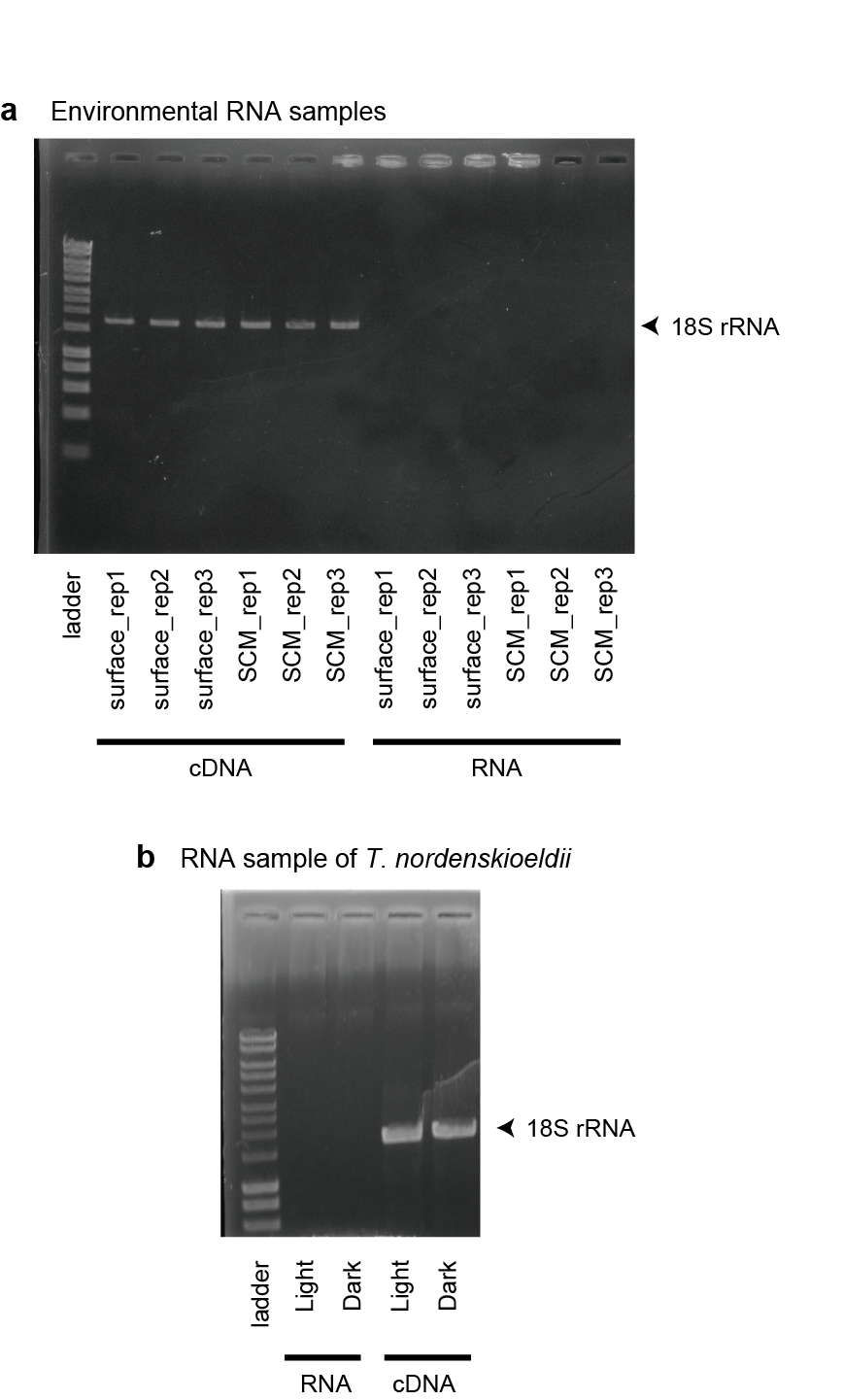


**Supplementary Figure S11. Gel image of electrophoresis to confirm DNA contamination**

(a) Gel image of electrophoresis using the environmental samples. Lane 1: ladder; Lane 2–7: PCR using cDNA as templates; Lane 8–13: PCR using RNA as templates. (b) Gel image of electrophoresis using the *T. nordenskioeldii* RNA sample. The gel images were not cropped. Lane 1: ladder; Lane 2–3: PCR using RNA as templates; Lane 4–5: PCR using cDNA as templates.
